# Utilization of Germinated Seeds as Functional Food Ingredients: Optimization of Nutrient Composition and Antioxidant Activity Evolution Based on the Germination Characteristics of Chinese Chestnut (*Castanea mollissima*)

**DOI:** 10.3390/foods13162605

**Published:** 2024-08-20

**Authors:** Junwei Yuan, Haifen Wang, Yunbin Jiang, Yuqian Jiang, Yao Tang, Xihong Li, Yuhua Zhao

**Affiliations:** 1 Chestnut Research Center, Hebei Normal University of Science and Technology, Qinhuangdao 066004, China; junweiyuan@126.com (J.Y.); yuanbinjiang@126.com (Y.J.); 2 College of Food Science and Technology, Hebei Normal University of Science and Technology, Qinhuangdao 066000, China; yuhuazhao@126.com; 3 College of Food Science and Engineering, Tianjin University of Science and Technology, Tianjin 300457, China; jiangyuqian16@163.com (Y.J.); tangyao886@hotmail.com (Y.T.); lixihong606@outlook.com (X.L.)

**Keywords:** *Castanea mollissima*, germination, functional properties, antioxidant capacity, sprout, cotyledon

## Abstract

The current study investigated the impact of germination duration on the functional components (vitamin C, γ-aminobutyric acid (GABA), polyphenols, flavonoids) and antioxidant activity of germs and cotyledons of the germinated Chinese chestnut (*Castanea mollissima*). We utilized seeds of the “Zaofeng” Chinese chestnut to germinate, and sowed the seeds in wet sand at 22 °C and 85% relative humidity. The germination rate, length, diameter, and fresh weight of the sprouts were investigated at 0, 2, 4, 6, 8, and 10 days after sowing, and the kinetic changes of amylose, amylopectin, sugar components, soluble protein, vitamin C, GABA, total phenols, flavonoids, and the DPPH and ABTS free radical scavenging activity in the germs and cotyledons were monitored, respectively. The findings revealed that the germination rate and germ biomass increased continuously during germination. The germination rate reached 90% on the 8th day after sowing. Germination reduced amylose in cotyledons from 42.3% to 34.2%, amylopectin from 42.9% to 25.8%, total sugar from 12.6% to 11.4%, and vitamin C from 1.45 mg/g to 0.77 mg/g. Meanwhile, soluble protein in the embryos rose from 0.31% to 0.60%, vitamin C from 21.1 to 29.4 mg/g, GABA from 0.49 to 1.68 mg/g, total flavonoids from 53.6 to 129.7 mg/g, and ABTS antioxidant activity from 1.52 to 3.27 μmol TE/g. The average contents of D-fructose, inositol, vitamin C, GABA, polyphenols, and flavonoids and the DPPH and ABTS antioxidant activity in germs were as high as 22.5, 6, 35, 7.5, 10, 20, and 10 and 20-fold those of cotyledons, respectively. Especially, the average content of glucose in germ was as high as 80-fold that of cotyledon. D-xylulose, D-galacturonic acid, and D-ribose were only found in germs, but not in cotyledons. Considering the germ biomass and functional components content, germs of Chinese chestnuts germinated at 22 °C for 8 days are considered the most suitable raw material for functional food products. In conclusion, controlled germination not only enhances the physicochemical and functional properties of Chinese chestnut germs but also reduces the caloric content and improves the nutritional composition of the cotyledons appropriately. Moreover, the comprehensive evaluation of compositional changes and functionality in the embryo and cotyledon of Chinese chestnuts will provide a solid foundation for subsequent functional food processing utilizing germinated Chinese chestnuts.

## 1. Introduction

The Chinese chestnut (*Castanea mollissima*) is a widely consumed food in numerous countries and serves as a significant source of starch. As a perennial crop, the Chinese chestnut is not only an important starch-based food, for its starch content is as high as 60–80%, but also a potential functional food because it is a rich source of bioactive compounds, including phenols, flavone, vitamin C, B vitamins, etc. [1]. China stands as the leading global producer of chestnuts. In 2019, the worldwide production of Chinese chestnuts totaled approximately 2.2 million tons, with China contributing 118,000 tons, representing over 90% of the global output [2]. However, Chinese chestnuts with elevated starch levels may increase the risk of colon cancer, hemorrhoids, diverticulosis, constipation, and allergic reactions in some consumers [1]. Presently, there is a growing consumer demand for natural and healthy foods, leading to an increasing popularity of germinated products among individuals seeking to enhance and sustain their health through dietary modifications [3].

Germination, serving as the initial transformation process [4], triggers a range of biochemical reactions such as hydrolysis, macromolecule biosynthesis, respiration, subcellular structures, etc., leading to the accumulation of diverse primary and secondary metabolites in seeds [5]. Following germination, enhancements in the nutritional profile, functional properties, and flavor of seeds can be achieved [6], potentially reducing the occurrence of allergic reactions [7]. As processing technologies advance and living standards improve, there is an increasing focus on dietary health and food safety. Plant-based products, enriched with balanced nutrition and abundant functional bioactive compounds obtained through germination, align with the evolving preferences of the consumer market and offer hundreds of millions of USD of large-scale market growth for the world [8].

Prior research has demonstrated that germination can diminish the levels of trypsin inhibitors, phytic acid, and other undesirable components, thereby mitigating issues related to indigestion, allergies, and other concerns [9]. Throughout the germination process, a cascade of alterations takes place in the nutritional constituents of grains, influencing the flavor and palatability following thermal processing [10]. When seeds germinate, starch granules are hydrolyzed by amylase and converted into dextrin and maltose, and then further decomposed into glucose by maltase. Glucose produced by hydrolysis is quickly transferred to hypocotyl for seed germination and growth. Protein is gradually decomposed into free amino acids, which are transported to the embryo to form the protoplasm of new cells [11]. Following germination, the starch content of various grains decreases, leading to reductions in peak viscosity, trough viscosity, and final viscosity during germination [12]. As grains germinate, lipase activation occurs, initiating the breakdown of stored fats within the seeds. The resulting fatty acids and other compounds engage in the active glyoxylate cycle during seed germination, providing energy for the growth and development of the sprout [13]. Studies have indicated a decrease in fat content in highland barley following germination [14]. Subsequent to germination, the fat contents of wheat, rice, oats, and corn decreased by 0.9%, 0.62%, 2.01%, and 0.87%, respectively [15]. Soybean germination typically results in an increase in protein content, possibly attributed to elevated levels of specific amino acids like lysine and tryptophan, or the reduction in dry weight due to the utilization of carbohydrates for respiration [16]. In the germination process of quinoa, there is an initial decline in protein content followed by an increase in the later stages of germination [17]. Conversely, a reduction in protein content is observed in brown rice germination, potentially resulting from the breakdown of soluble proteins to facilitate substance metabolism or influenced by protein content changes due to sample preparation techniques [18]. The protein content of brown rice notably decreases with prolonged germination time and increased germination temperature, with a 6.35% reduction observed at 35 °C for 48 h [19]. The germination process of grains also influences the nutrient composition, including minerals and vitamins. Following high-pressure germination of brown rice, the vitamin E content rose to 4.34 mg/100 g from 2.06 mg/100 g [20]. The enhanced vitamin E content during germination is primarily attributed to the synthesis of tocopherol and tocotrienol. Post-germination, the vitamin B_2_ content in black beans and soybeans has been found to be twice as high as that in ungerminated soybeans and black beans [21]. It was observed that vitamin C was present in both mung bean sprouts and brown bean sprouts, while it was undetectable in the dry seeds of these plants. This confirms the ability of plants to synthesize vitamin C from glucose, mannose, and galactose [22]. During germination, the enhancement of vitamin C levels is facilitated by the enzymatic breakdown of starch by amylase, leading to increased availability of glucose for vitamin C synthesis [23].

GABA, known for its excellent water solubility, thermal stability, and food safety, finds wide applications in the production of beverages and various food products [24]. Baranzelli et al. found that the GABA content of germinated wheat was approximately 2.3 times higher compared to pre-germination levels [25]. In the case of rice germinated in darkness, an increase in germination time corresponded to higher yields of glutamic acid and GABA, with the GABA content reaching 34.28 mg/100 g after 48 h of germination [26]. Proteolytic enzymes are activated during germination, breaking down proteins into free amino acids, notably glutamic acid, which are subsequently converted into GABA compounds [27]. In Chinese wild rice, total, free, and bound phenolic levels increased significantly by 57.85%, 46.60%, and 93.58%, respectively, at the completion of germination compared to raw seeds [28]. Germinated cotyledons and sprouts exhibit various bioactivities including antibacterial, anti-inflammatory, antidiabetic, and antioxidant properties [29]. Through 2,2-diphenyl-1-picrylhydrazyl (DPPH) and 2,2-azino-bis-3-ethylbenzthioazo line-6-sulfonic acid (ABTS) assays, it was determined that germination notably enhances the antioxidant activity of canary seeds [30]. Germinated edible seeds and sprouts can serve as functional foods for the prevention of specific chronic diseases due to their abundance of natural bioactive compounds [31], offering a promising avenue for developing green foods rich in bioactive compounds and antioxidant activity [32]. Soybean sprouts exhibit a broader array of functional compounds compared to non-germinated soybean seeds, with significant increases in isoflavones, L-ascorbic acid, and phenolic compounds observed during the germination process [33]. Usually, seed germination is carried out in a high-humidity environment. If the conditions are not well controlled, mold will easily breed, which will not only affect the germination rate, but also cause food safety hazards [34]. Presently, research predominantly concentrates on annual crop seeds rather than perennial tree seeds [35].

The Chinese chestnut is rich in bioactive compounds such as polyphenols, flavonoids, and saponins, which exhibit physiological effects including antioxidative properties, immune enhancement, blood sugar and lipid reduction, cardiovascular disease prevention and treatment, and overall health maintenance. Germination serves as a simple and effective method to enhance its characteristics. It facilitates the conversion of macromolecular substances into easily absorbable micromolecular compounds, while reducing the content of anti-nutritional factors. However, current scholarly research on germinated Chinese chestnuts primarily focuses on breeding and cultivation aspects, with limited exploration into germinated Chinese chestnuts as a functional food. The current study investigated the impact of germination duration on the morphology, biomass, nutritional composition (carbohydrates, proteins, vitamin C), functional bioactive components (GABA, total polyphenols, and flavonoids), and antioxidant activity (DPPH and ABTS radical scavenging activity) of various sections of the Chinese chestnut. Furthermore, the study preliminarily explored the mechanisms underlying the enhancement of nutritional and functional bioactive components in Chinese chestnuts through germination. These findings aim to offer valuable theoretical insights for the comprehensive investigation of processing germinated Chinese chestnuts.

## 2. Materials and Methods

### 2.1. Materials

We selected seeds of the Chinese chestnut (*Castanea mollissima*) cultivar “Zaofeng” harvested from robust trees in the primary Chinese chestnut-producing area of Qianxi County, Tangshan City, Hebei Province, China, on 21 September 2023. Subsequently, the seeds were stored at −3 °C until further processing.

### 2.2. Germination and Sample Preparation

On 1 March 2024, the Chinese chestnut seeds were processed according to a previously documented method [36], with slight adjustments. Initially, uniform Chinese chestnut seeds were washed thrice with sterile water and air-dried. Subsequently, the seeds were evenly distributed on a tray (50 cm × 30 cm × 15 cm) filled with sterilized sand mixed with sterile water, burying them under a layer of wet sand approximately 5 cm thick. These trays were then positioned at 22 °C and 85% relative humidity. The germination rate of the seeds was recorded, and the length, diameter, and fresh weight of the sprouts were measured at 2, 4, 6, 8, and 10 days post-sowing, and the germination rate was determined by calculating the ratio of germinated seeds to the total number of seeds in each treatment [33]. Ten seeds per replicate were collected at intervals of 0, 2, 4, 6, 8, and 10 days post-sowing. Following the removal of seed coats, the cotyledons and sprouts were chopped, freeze-dried, and subsequently ground for further physiological analyses. Each treatment was conducted in triplicate, with each replicate consisting of 10 seeds.

### 2.3. Length, Diameter, and Fresh Weight of Sprouts

Thirty germinated Chinese chestnut seeds were randomly chosen for analysis. The length of the Chinese chestnut sprouts was measured using a soft ruler with a minimum scale of 0.01 cm, while the sprout diameter was measured using a vernier caliper with a minimum scale of 0.01 mm. Additionally, the fresh weight of the sprouts was determined with a balance that had a minimum scale of 0.01 g.

### 2.4. Determination of Amylose and Amylopectin

Following the method outlined by Cruz et al. [37] with appropriate modifications, 50 mg of the sample was weighed and combined with 1 mL of 80% ethanol for extraction in a water bath at 80 °C for 30 min. After centrifugation and removal of the supernatant, the precipitate was retained and treated with 1 mL of ether for degreasing. Subsequent centrifugation and removal of the supernatant left the precipitate for drying. The dried precipitate was then dissolved in 1 mL of 0.5 mol/L KOH solution and heated in a water bath at 90 °C for 10 min, and the supernatant was collected after cooling. Subsequently, 500 μL of the test liquid was transferred into a 10 mL centrifuge tube, followed by the addition of 500 μL of a 0.2 mol/L HCl solution, 2.0 mL of distilled water, and 200 μL of iodine reagent. Absorbance readings at 475 nm, 615 nm, 550 nm, and 695 nm wavelengths (UV-Vis spectrophotometer; UT-1901; METASH, Instrument Co., Ltd., Shanghai, China) were recorded as A_475_, A_615_, A_550_, and A_695_, respectively. The calculations involved determining ΔA_Amylose_ = A_475_ − A_615_ and Δ_Amylopectin_ = A_550_ − A_695_. The obtained values were compared against standard curves for amylose and amylopectin solutions, respectively. Starch content was expressed as mg of amylose and amylopectin per gram of dry weight of the sample (mg/g DW).

### 2.5. Determination of Sugar Components

The sugar components were analyzed using the GC-MS method outlined by Sun et al. [38] with certain adjustments. Initially, 20 mg of powder was weighed and combined with 500 μL of methanol–isopropanol–water (3:3:2, *v*/*v*) extraction solution. The mixture was vortexed for 3 min and subjected to ultrasonic treatment in ice water for 30 min. Subsequently, centrifugation at 4 °C and 13,000× *g* for 5 min was conducted. Following the absorption of 50 μL of the supernatant, 20 μL of internal standard solution with a mass concentration of 1000 μg/mL was added, and the solution was dried using nitrogen and a freeze-dryer. Further steps involved the addition of 100 μL of methoxyamine salt pyridine (15 mg/mL), followed by an incubation at 37 °C for 2 h. Subsequently, 100 μL of bis(trimethylsilyl)trifluoro-acetamide was added, and the solution was incubated at 37 °C for 30 min to obtain the extract. We transferred 50 μL of the extract and diluted it to 1 mL with n-hexane. We stored the diluted extract in an amber injection vial for further analysis. The standard solution was prepared using D-[1,2,3,4,5,5′-2H6] ribose with a mass concentration of 1000 μg/mL.

### 2.6. Determination of Vitamin C Content

Vitamin C was quantified following the method outlined by Ye et al. [39] with certain modifications. Initially, 50 mg of the sample was weighed and combined with 500 μL of 1 mol/L HClO_4_. The mixture was ground in an ice bath using a mortar and then centrifuged at 13,000× *g* at 4 °C for 5 min. The resulting supernatant was mixed with 0.1 mol/L 4-(2-hydroxyethyl)-1-piperazineethanesulfonic acid (HEPES)/KOH buffer at pH 7.0 in a 1:5 ratio (buffer/extract) and neutralized to pH 5.6 with 1 mol/L K_2_CO_3_ before undergoing centrifugation as previously described. The supernatant was divided into two tubes, with one tube treated with 1.5 mL of ascorbic acid oxidase (100 U/mL, Sigma, St. Louis, MO, USA) and the other serving as a control with the addition of the same volume of distilled water. After a 30 min incubation period, vitamin C content was determined by measuring the difference in A_265_ nm between the two tubes.

### 2.7. Determination of GABA Content

The GABA content was analyzed using an HPLC-MS method as detailed by Zhao et al. [40], with minor adjustments. Initially, 200 mg of the sample was extracted with 1 mL of hydrochloric acid solution (0.1 mol/L) at 70 °C for 1 h, followed by centrifugation at 12,000× *g* for 10 min. A 10 μL aliquot of the supernatant was transferred to a derivatization tube and mixed with 70 μL of accqtag ultraborate buffer and 20 μL of accqtag reagent. The mixture was vortexed and heated at 55 °C for 10 min, then cooled for subsequent analysis. Quantification of GABA was based on a standard curve of GABA and expressed as mg/kg dry weight (DW).

### 2.8. Determination of Total Phenolic Content

The total phenolic content of the samples was determined using the Folin–Ciocalteu spectrophotometric method as Zhao et al. [41] outlined, with some modifications. Initially, 500 μL of each sample was aliquoted into 10 mL centrifuge tubes and mixed with 3.00 mL of distilled water and 250 μL of Folin–Ciocalteu phenolic reagent, with the reaction allowed to proceed for 5 min. Subsequently, 750 μL of 7% (*w*/*v*) Na_2_CO_3_ was added, and after 1 h at 20 °C, absorbance readings were taken at 765 nm using a UV-Vis spectrophotometer (UT-1901; METASH, instrument Co., Ltd., Shanghai, China). The total phenolic content was determined using a standard curve generated from a gallic acid (GAE) solution. The results were expressed as mg gallic acid equivalents (GAE) per gram of dry weight of the sample (mg GAE/g DW).

### 2.9. Determination of Total Flavonoid Content

In accordance with the method outlined by Farhadi et al. [42] with appropriate modifications, the sample extract (250 µL) was mixed with distilled water (1250 µL), followed by the addition of 5% Na_2_NO_3_ solution (75 µL). After a 5 min incubation period, 10% AlCl_3_·6H_2_O (150 µL) was introduced into the reaction mixture. Subsequently, 500 µL of 1 mol/L NaOH and 275 µL of distilled water were added. The absorbance of the reaction mixture was then measured at 510 nm using a UV-Vis spectrophotometer (UT-1901; METASH, instrument Co., Ltd., Shanghai, China). The total flavonoid content (TFC) value of the sample was determined using a standard curve generated from a catechin (GAE) solution. The results were expressed as mg catechin equivalents per gram of dry sample (mg CAE/g DW).

### 2.10. DPPH Radical Scavenging Activity

The DPPH radical scavenging activity of the sample was assessed following the method described by Farhadi et al. [43], with suitable modifications. Initially, 100 μL of the methanol extract from each sample was placed into 10 mL centrifuge tubes and combined with 3.9 mL of a 0.1 mmol/L methanol solution of DPPH. The mixture was thoroughly mixed and then incubated in the dark for 30 min. Subsequently, the absorbance was measured at 517 nm using a UV-Vis spectrophotometer (UT-1901; METASH, instrument Co., Ltd., Shanghai, China). The radical scavenging activity was determined using a standard curve generated from a Trolox solution. The results were expressed as mg Trolox equivalents per gram of sample (mg TE/g DW).

### 2.11. ABTS Radical Scavenging Activity

The ABTS radical cation scavenging activity was determined following the method outlined by Chang et al. [44], with appropriate modifications. Initially, 10 mL of the ABTS stock solution was diluted with 25 mL of 0.325 mol/L phosphate buffer and 65 mL of Milli-Q water prior to analysis. Subsequently, 100 μL of the methanol extract from each sample was combined with 1.9 mL of 0.325 mol/L phosphate buffer and 2.0 mL of the diluted ABTS radical cation solution. The mixture was thoroughly mixed and incubated in the dark for 1 h. Absorbance readings were taken at 734 nm using a UV-Vis spectrophotometer (UT-1901; METASH, instrument Co., Ltd., Shanghai, China). The radical scavenging activity was determined using a standard curve generated from a Trolox solution, and the results were expressed as mg Trolox equivalents per gram of dry weight (mg TE/g DW).

### 2.12. Statistical Analysis

All experiments were conducted in triplicate, and the results are reported as the average values with standard errors. Statistical analyses were performed using SPSS 22.0 (SPSS Inc., Chicago, IL, USA), while graphing was carried out with Origin 2023 (Microcal Software, Northampton, MA, USA). Duncan’s multiple range tests were employed for the ANOVA, and a significance level of *p* < 0.05 was considered statistically significant.

## 3. Results

### 3.1. Effect of Germination Time on the Growth of Chinese Chestnut Sprouts

**Figure 1:**
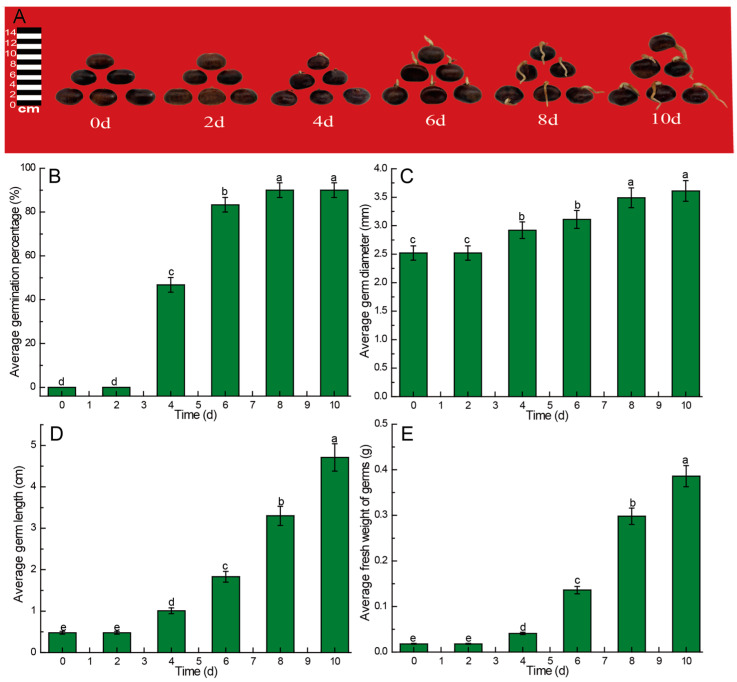
Comparison of morphology (**A**), average germination rate (**B**), germ diameter (**C**), germ length (**D**), and germ fresh weight (**E**) of Chinese chestnuts during germination. Values in the figures are shown as the means ± standard error (*n* = 3). Vertical bars represent the standard errors of the means. Different letters represent significant differences among treatments for each sampling time at *p* ≤ 0.05.

In general, the germination process of the Chinese chestnut as a perennial tree will last for approximately one week. Therefore, we monitored this germination process for 10 days. The changes in germination rate, length, diameter, and fresh weight of Chinese chestnut sprouts during germination at days 0, 2, 4, 6, 8, and 10 are depicted in Figure 1. The Chinese chestnut sprouts in this study exhibited a growth pattern characterized by an initial slow phase followed by rapid growth. By the 8th day post-sowing, the sprouts began to show lignification throughout the germination process, rendering them unsuitable for subsequent food processing (Figure 1A). Germination of the Chinese chestnut seeds commenced on the 3rd day, with the germination rate reaching 46.67% and 83.33% on the 4th and 6th days, respectively, before peaking at 90% on the 8th day post-sowing (Figure 1B). In comparison to other parameters, the growth in germ diameter exhibited a relatively gradual increase, reaching 2.92 mm and 3.11 mm on the 4th and 6th days, respectively. Although there was a significant increase compared to the initial value (*p* < 0.05), no significant difference was observed between the subsequent time points (*p* > 0.05). The increase in germ diameter was no longer statistically significant (*p* < 0.05) after 8 days of sowing (Figure 1C). The average germ length exhibited a steady increase throughout the germination period. Over the course of 10 days, the average germ length reached 4.71 cm (Figure 1D). Similarly, the fresh weight of the germs followed a pattern similar to the average germ length during germination. The average fresh weight of the germs increased to 0.298 g and 0.386 g on the 8th and 10th days, respectively, representing 4.26% and 5.51% of the average fresh weight of the entire chestnut kernel (Figure 1E).

### 3.2. Effects of Germination Time on Nutrient Composition in Different Parts of Germinated Chinese Chestnuts

#### 3.2.1. Amylose and Amylopectin

**Figure 2:**
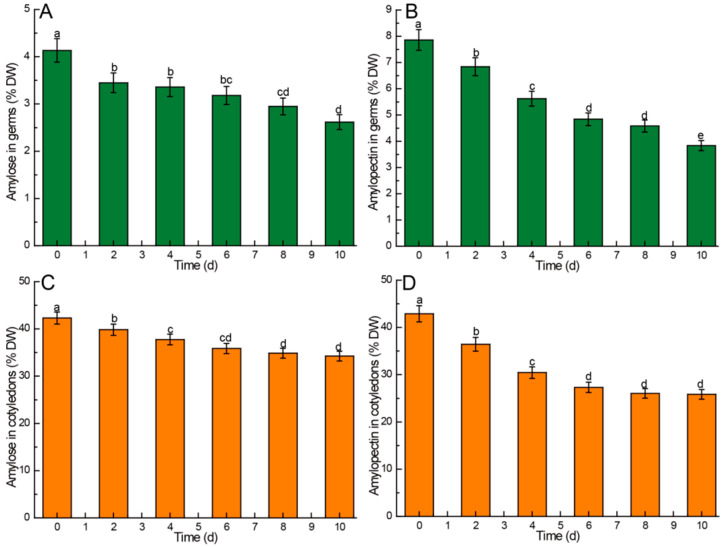
Comparison of amylose and amylopectin in different parts of Chinese chestnuts during germination. (**A**) amylose in germs, (**B**) amylopectin in germs, (**C**) amylose in cotyledons, (**D**) amylopectin in cotyledons. Values in the figures are shown as the means ± standard error (*n* = 3). Vertical bars represent the standard errors of the means. Different letters represent significant differences among treatments for each sampling time at *p* ≤ 0.05.

The germination process of Chinese chestnuts is a process of consuming energy, and starch, as the most important energy source in Chinese chestnut seeds, will be consumed during the germination process. The comparison of amylose and amylopectin levels in different parts of Chinese chestnuts during germination is illustrated in Figure 2. The findings revealed a gradual decrease in the contents of amylose and amylopectin in the various parts of Chinese chestnuts (germ and cotyledon) throughout the germination process. The content of amylose in germ decreased from 4.13% to 2.62%, amylopectin from 7.86% to 3.84%, amylose in cotyledon from 42.30% to 34.24%, and amylopectin from 42.89% to 25.84%. Notably, the cotyledons exhibited significantly higher levels of both amylose and amylopectin compared to the germ under the same experimental conditions. Furthermore, the amylopectin content in the germ was significantly higher than the amylose content under identical conditions (*p* < 0.05). In comparison to the amylose content in cotyledons, the amylose content in the germ exhibited a more rapid decrease. Additionally, both the amylopectin content in cotyledons and germs declined at a faster rate than that of amylose, suggesting that amylopectin in both cotyledons and germs was more susceptible to hydrolysis compared to amylose during germination.

Although the amylose content in the germ decreases continuously during germination, the total amount of amylose in the germ does not decrease but rather increases to some extent, despite the ongoing growth of the germ. On the 2nd, 4th, and 6th days following sowing, the amylose content in the germ showed a significant decrease compared to the initial value (*p* < 0.05), with no significant difference observed between these time points (*p* > 0.05). By the 10th day, a significant decrease in amylose content was noted (Figure 2A). Conversely, the amylopectin content in the germ exhibited a significant decrease (*p* < 0.05) during the initial 6 days post-sowing, with a slowdown in the rate of decline observed in the later stages (8th–10th days) of germination (Figure 2B). The amylose content in cotyledons decreased from 42.30% to 37.75% (*p* < 0.05) during the initial four days post-sowing, with a deceleration in the rate of decline (from 37.75% to 34.24%) observed in the later stage (6th–10th days) of germination (Figure 2C). Similarly, the amylopectin content in cotyledons decreased from 42.89% to 27.32% (*p* < 0.05) in the first six days following sowing, with a reduction in the rate of decline (from 27.32% to 25.84%) noted in the later stages of germination (Figure 2D).

#### 3.2.2. Sugar Components

During germination, the starch of Chinese chestnut hydrolysis is transformed into various sugar components. The comparison of sugar components in different parts of germinated Chinese chestnuts during germination is presented in Table 1. The findings revealed that the sugar content in the germ was significantly higher than that in the cotyledon (*p* < 0.05). The predominant sugars in the chestnut germ were identified as sucrose, glucose, D-fructose, and inositol, collectively constituting over 98% of the total sugar content. In contrast, sugars in the cotyledons were predominantly in the form of sucrose, accounting for over 92% of the total sugar content. Notably, D-xylulose, D-galacturonic acid, and D-ribose were exclusively present in the chestnut embryo and were absent in the cotyledons.

During the germination process, the total soluble sugar content in the germ exhibited a decreasing trend, whereas the content in the cotyledons remained relatively stable in the early stages of germination (up to the first 8 days) before a significant decrease (*p* < 0.05) was observed on the 10th day. The sucrose content in the cotyledons was notably higher than in the embryos. In the germ, the sucrose content displayed a noticeable downward trend during germination, with a slower rate of decline in the early stages (first 2 days), an accelerated decline in the middle stages (4th day to 8th day), and a deceleration in the later stages (8th day to 10th day). In contrast to sucrose, the levels of glucose, D-fructose, and inositol in the germs were significantly higher than those in the cotyledons. In the cotyledons, the glucose content exhibited a continuous increase leading up to the 6th day before germination, reaching its peak at this point, and subsequently decreasing during the later stage (6th to 8th days) of germination. Conversely, the glucose content in the germ displayed a decreasing trend throughout germination, with a gradual decline in the initial 8 days followed by an accelerated decrease after the 8th day. The trends observed for fructose were similar to those of glucose, with the relative content and changes during germination in both cotyledons and embryos aligning closely with the patterns observed for glucose. In the cotyledons, the inositol content exhibited a fluctuating pattern during germination, gradually decreasing initially and then declining rapidly in the germ.

#### 3.2.3. Soluble Protein

**Figure 3:**
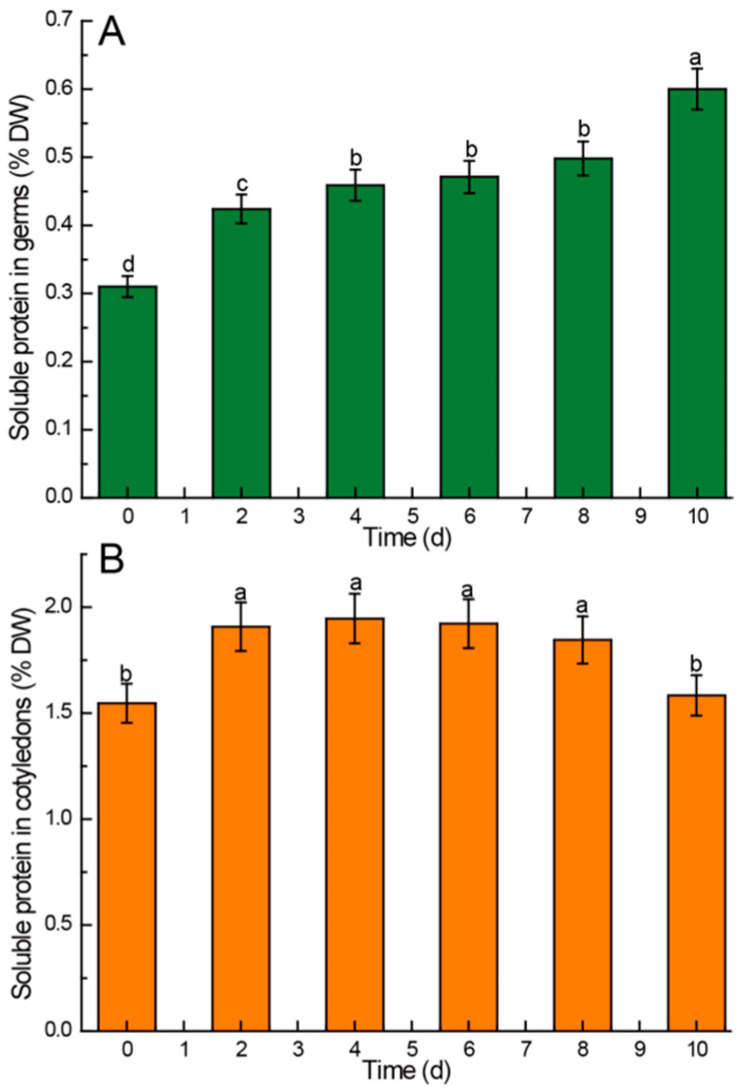
Comparison of soluble protein content in different parts of Chinese chestnuts during germination. (**A**) soluble protein in germs, (**B**) soluble protein in cotyledons. Values in the figures are shown as the means ± standard error (*n* = 3). Vertical bars represent the standard errors of the means. Different letters represent significant differences among treatments for each sampling time at *p* ≤ 0.05.

During the germination of Chinese chestnuts, many nutrients need to be transported, so the soluble protein in seeds will change greatly. The findings depicted in Figure 3 revealed that the soluble protein content in the germs of Chinese chestnuts was notably higher than that in the cotyledons. During germination, the soluble protein content in the germ exhibited a rapid increase in the initial 4 days, followed by a gradual slowing of change from the 4th day to the 8th day, and a subsequent rapid increase after 8 days (Figure 3A). In contrast, the soluble protein content in the cotyledons demonstrated a significant increase (*p* < 0.05) in the first 2 days, with no statistically significant difference observed from the second day to the eighth day. However, a significant decrease (*p* < 0.05) was noted after 8 days of germination (Figure 3B).

#### 3.2.4. Vitamin C

**Figure 4:**
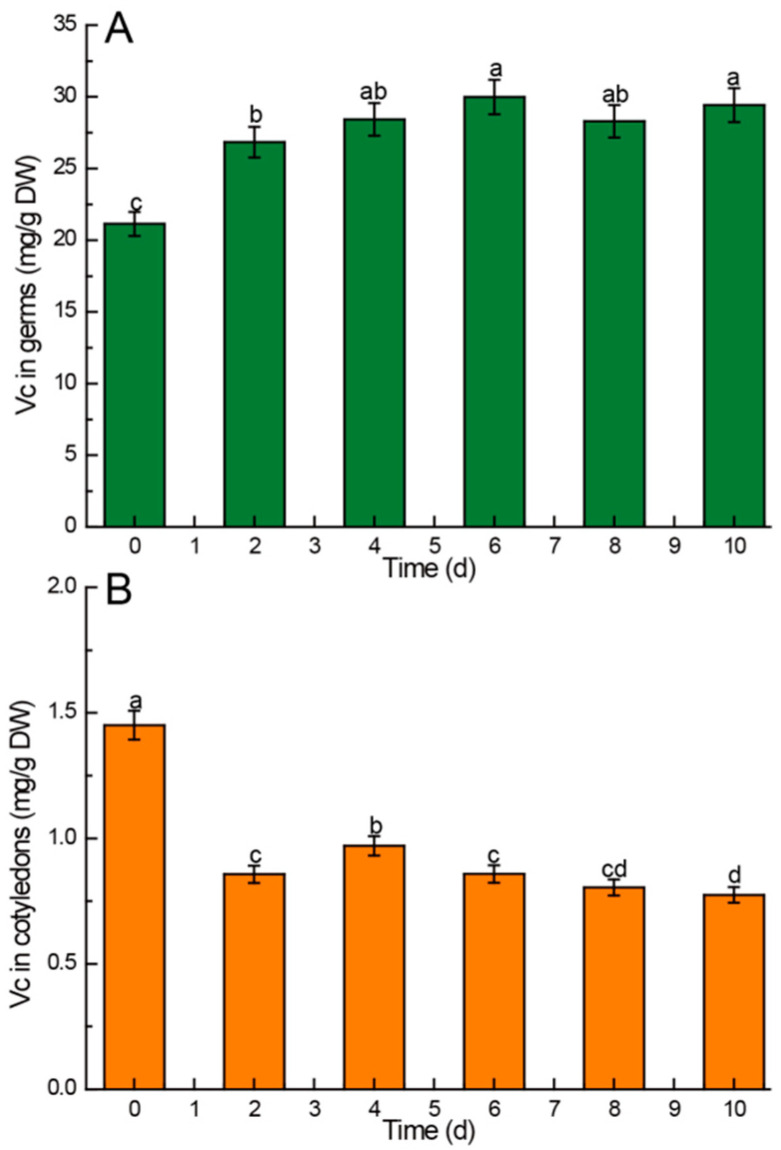
Comparison of vitamin C content in different parts of Chinese chestnuts during germination. (**A**) vitamin C in germs, (**B**) vitamin C in cotyledons. Values in the figures are shown as the means ± standard error (n = 3). Vertical bars represent the standard errors of the means. Different letters represent significant differences among treatments for each sampling time at *p* ≤ 0.05.

Vitamin C is closely related to sugar metabolism, so germination will lead to a change in vitamin content in Chinese chestnuts. The vitamin C content in the germs of Chinese chestnuts was markedly higher than that in the cotyledons, as illustrated in Figure 4. In the Chinese chestnut germs, the ascorbic acid content exhibited a significant increase (*p* < 0.05) in the first 4 days of germination. Subsequently, although there were slight fluctuations in the content after 4 days, the differences reached a level of statistical significance (*p* > 0.05) (Figure 4A). Conversely, the vitamin C content in the cotyledons experienced the most substantial decrease in the initial 4 days (*p* < 0.05). Although there was a slight increase on the 4th day, the overall trend indicated a decline in vitamin C content throughout the entire germination process.

### 3.3. Effects of Germination Time on Functional Components Content in Different Parts of Germinated Chinese Chestnuts

#### 3.3.1. GABA

**Figure 5:**
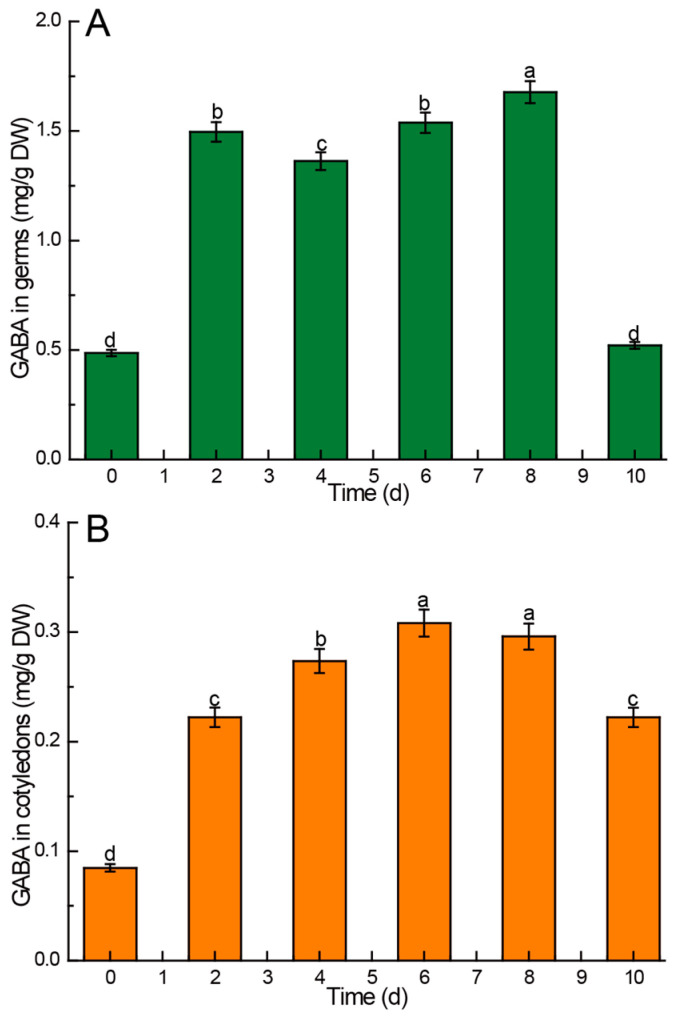
Comparison of γ-aminobutyric acid (GABA) content in different parts of Chinese chestnuts during germination. (**A**) GABA in germs, (**B**) GABA in cotyledons. Values in the figures are shown as the means ± standard error (n = 3). Vertical bars represent the standard errors of the means. Different letters represent significant differences among treatments for each sampling time at *p* ≤ 0.05.

The findings revealed a significantly higher content of GABA in the germs both before and after germination compared to the cotyledons, as depicted in Figure 5. With the exception of the 10th day after sowing, the post-germination GABA levels in the germs were consistently higher than those observed pre-germination. In the germs, the GABA content was lower on the 4th day compared to the 2nd day, but it steadily increased on the 6th day, reaching a peak of 1.677 mg/g DW on the 8th day, before rapidly declining to pre-germination levels on the 10th day (Figure 5A). Similarly, the GABA content in the cotyledons of Chinese chestnuts post-germination exceeded that observed pre-germination. The GABA content in the cotyledons increased continuously in the first six days after sowing, reaching the peak value of 0.308 mg/g DW on the sixth day, followed by a gradual decrease (Figure 5B).

#### 3.3.2. Total Phenols and Flavonoids

**Figure 6:**
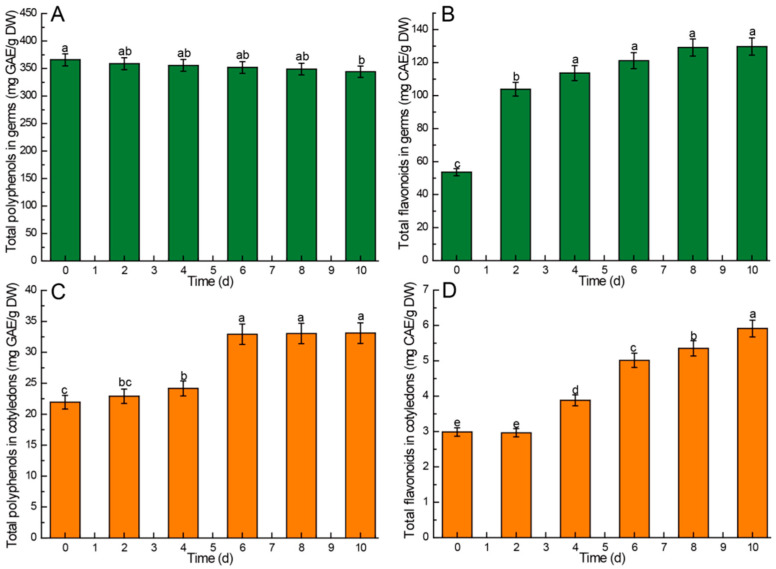
Comparison of total polyphenols and flavonoids contents in different parts of Chinese chestnuts during germination. (**A**) total polyphenols in germs, (**B**) total flavonoids in germs, (**C**) total polyphenols in cotyledons, (**D**) total flavonoids in cotyledons. Values in the figures are shown as the means ± standard error (n = 3). Vertical bars represent the standard errors of the means. Different letters represent significant differences among treatments for each sampling time at *p* ≤ 0.05.

The results indicated that the content of total phenols and flavonoids in the germs was notably higher than that in the cotyledons. Moreover, the total phenol content in both cotyledons and germs surpassed that of total flavonoids, as shown in Figure 6. In the germs, the content of polyphenols exhibited a slow decrease during germination, with the extent of decline not reaching statistical significance (*p* > 0.05) (Figure 6A). Conversely, the flavonoid content in the germs increased initially and then stabilized, particularly from day 4 to day 10. Although there was an increase in content during this period, the differences were not statistically significant (*p* > 0.05) (Figure 6B). In the cotyledons, the polyphenol content increased gradually in the first four days, followed by a rapid increase on the sixth day, with no significant differences (*p* > 0.05) observed in the subsequent days (Figure 6C). The flavonoid content in the cotyledons did not exhibit a significant increase (*p* > 0.05) in the initial 2 days; however, the growth rate accelerated after the 4th day, leading to a significant increase (*p* < 0.05) (Figure 6D).

### 3.4. Effects of Germination Time on Antioxidant Activity of Different Parts of Germinated Chinese Chestnuts

**Figure 7:**
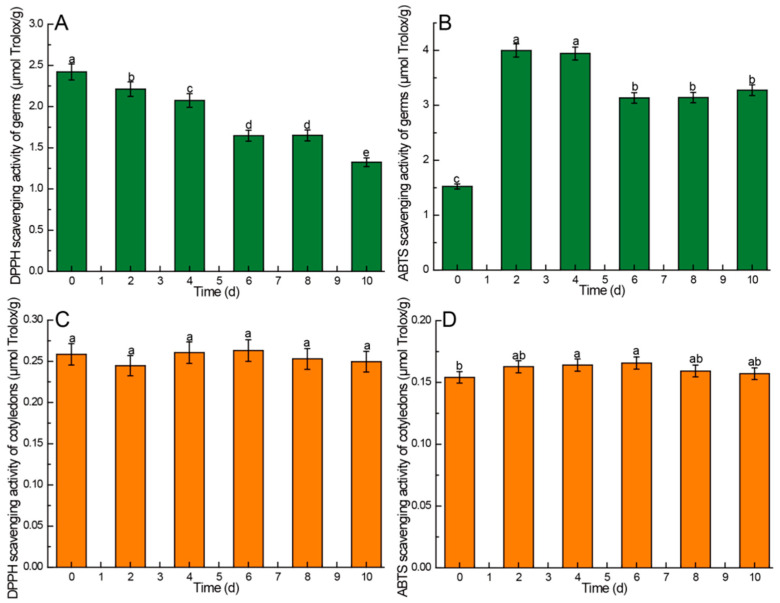
Comparison of DPPH and ABTS antioxidant activity of different parts of Chinese chestnut during germination. (**A**) DPPH scavenging activity of germs, (**B**) ABTS scavenging activity of germs, (**C**) DPPH scavenging activity of cotyledons, (**D**) ABTS scavenging activity of cotyledons. Values in the figures are shown as the means ± standard error (*n* = 3). Vertical bars represent the standard errors of the means. Different letters represent significant differences among treatments for each sampling time at *p* ≤ 0.05.

The results revealed a significantly higher DPPH and ABTS free radical scavenging activity in the germs compared to the cotyledons. Additionally, the DPPH free radical scavenging activity in both germs and cotyledons exceeded that of ABTS, as illustrated in Figure 7. In the germs, the DPPH free radical scavenging activity exhibited a decreasing trend during germination, with a faster decline in the initial 6 days. There were no significant differences observed on the 6th and 8th days (*p* > 0.05), but a significant decrease was noted on the 10th day (*p* < 0.05) (Figure 7A). The ABTS free radical scavenging activity in the germs post-germination surpassed that observed pre-germination, peaking on the 2nd and 4th days before decreasing in the subsequent days. However, it remained significantly higher than pre-germination levels (*p* < 0.05) (Figure 7B). In contrast, the DPPH free radical scavenging activity of cotyledons displayed varied values throughout the germination process, yet the differences were not statistically significant (*p* > 0.05) (Figure 7C). The ABTS free radical scavenging activity in cotyledons increased gradually in the initial 6 days, reaching its peak on the 6th day before declining in the following days; however, the differences were not significant (*p* > 0.05) (Figure 7C).

## 4. Discussion

In the current study, the impact of germination duration at 22 °C on the levels of starch, sugar components, soluble sugars, soluble proteins, vitamin C, GABA, polyphenols, flavonoids, and in vitro antioxidant activity in Chinese chestnut germs and cotyledons was investigated. The Chinese chestnut variety utilized in this study was a local genotype known as “Zaofeng”. This particular genotype was selected due to its high popularity, widespread consumption, and the preference among local residents for its use for food processing purposes as a raw material [45].

### 4.1. Kinetic Changes of Biomass Growth of Germ of Chinese Chestnut during Germination

In the present study, Chinese chestnuts commenced germination on the 3rd day, with the germination rate reaching 46.67% and 83.33% on the 4th and 6th days, respectively, culminating in a peak rate of 90% after 8 days of sowing. Post 10 days of sowing, the germs exhibited lignification, which was not conducive to subsequent processing. Therefore, we only chose the first 10 days as the scope of our study, although the growth rate of germ length and fresh weight gradually slowed down after 10 days. The growth pattern observed in the Chinese chestnut germs in this study aligned closely with previous research indicating a “slow-quick-slow” pattern in radicle length growth at 30 °C [46]. This is mainly because the growth of the germ is divided into three stages. (1) Cell division stage: at this stage, the number of cells is large but the volume is small, so the growth is slow. This is because the cells are in a period of division. Although the number of cells is increasing, the volume of each cell is relatively small, which leads to a slow overall growth rate. (2) Cell elongation stage: with the growth of cells, they enter the elongation stage. Due to the appearance and continuous elongation of vacuoles, the cell volume increases rapidly, so the growth rate is accelerated. This stage is a period of rapid increase in cell volume, which obviously accelerates the overall growth rate. (3) Cell differentiation stage: when the cell enters the differentiation stage, the volume is basically fixed, so the growth is extremely slow or even stopped. At this stage, the cell volume no longer changes significantly, and the growth rate slows down again until it finally stops growing. This S-shaped curve growth model is not only suitable for the germ, but is also suitable for the growth process of many plants, which reflects the universal law of biological growth in nature [47].

Moreover, the growth trend exhibited by Chinese chestnuts in this study indicated that the most rapid growth occurred between days 6 and 8 of germination at 22 °C.

### 4.2. Kinetic Changes of Amylose and Amylopectin in Chinese Chestnuts during Germination

Starch, comprising amylose and amylopectin, serves as the primary carbohydrate reserve in Chinese chestnuts. During germination, optimal temperature and moisture conditions break seed dormancy, triggering starch hydrolase activity. Consequently, total starch, amylose, and amylopectin in the endosperm gradually disperse and are enzymatically decomposed for seed respiration and metabolism, leading to a substantial decrease in total starch content [48]. This trend aligns with the findings of Quek et al. [49]. The higher amylose and amylopectin levels observed in cotyledons compared to germs can be attributed to cotyledons serving as the primary energy storage organs in Chinese chestnuts, where energy is predominantly stored in the form of starch [50]. Furthermore, there was a significantly higher amylopectin content in germs compared to amylose. In the process of germination, amylopectin decreased more than amylose in both germ and cotyledon. These results all suggest that amylopectin is more readily hydrolyzed than amylose [51].

### 4.3. Kinetic Changes of Sugar Components in Chinese Chestnuts during Germination

Glucose and sucrose serve a dual function in providing energy and signaling within plants. Soluble sugars, through their activation or inhibition of crucial processes, can have varying effects on germination. It is established that glucose within plant tissues can stimulate cell division, while sucrose is advantageous for differentiation and maturation processes [52].

In terms of sucrose content, cotyledons exhibited significantly higher levels compared to germs. The sucrose content in germs displayed a noticeable downward trend during germination. In contrast to sucrose, the concentrations of glucose, D-fructose, and inositol in germs were substantially higher than those in cotyledons. This disparity can be attributed to elevated levels of glucose, D-fructose, and inositol promoting accelerated mitosis in germs [53].

The glucose, D-fructose, and inositol contents in germs demonstrated a declining trend during germination. This clarifies that the consumption rate of glucose, D-fructose, and inositol exceeds the production rate during germination. The levels of glucose and D-fructose in cotyledons exhibited a steady increase until the 6th day preceding germination, possibly attributed to sugars being released during the germination process [54]. However, from 6 to 10 days after sowing, the decrease in glucose and D-fructose in cotyledons indicates that the consumption rate exceeds the production rate at this stage [55].

D-xylulose, D-galacturonic acid, and D-ribose are exclusively present in the embryo of Chinese chestnuts, not in the cotyledons. This finding indicates that these three sugars are characteristic sugar components of the Chinese chestnut germ.

### 4.4. Kinetic Changes of Soluble Protein in Chinese Chestnuts during Germination

The findings revealed a notably higher soluble protein content in Chinese chestnut germs compared to cotyledons. This discrepancy can be attributed to the fact that more active proteins are needed in the germ to transport more nutrients for germination during seed germination compared to cotyledons [56]. During the whole germination process, the content of soluble protein in the germ increased continuously. This shows that germination changes the existing form of protein in germ, and more proteins exist in soluble form.

On the other hand, the content of soluble protein in cotyledons increased significantly in the first 2 days, indicating that a large amount of soluble protein was needed to transport nutrients in cotyledons at the initial stage of germination. The content of soluble protein in cotyledons in the middle stage of germination remained relatively stable. The significant decrease in soluble protein content in cotyledons at the late stage of germination indicates that the transport of nutrients between cotyledons and embryos is reduced at this time. These results are consistent with the research findings on germinated brown rice by Cornejo et al. [3].

### 4.5. Kinetic Changes of Vitamin C in Chinese Chestnuts during Germination

Vitamin C, a glucose derivative resulting from strong oxidation, is a vital nutrient essential for maintaining normal human physiological functions. Research has demonstrated that plants can synthesize vitamin C from glucose, mannose, and galactose. Therefore, during germination, the elevation in vitamin C levels is driven by the enzymatic hydrolysis of starch by amylase, enhancing the availability of glucose for vitamin C biosynthesis. The increase in glucose content contributes to the rise in vitamin C content. This elucidates the finding that the ascorbic acid content in germs was significantly higher than that in cotyledons in the present study [57].

Vitamin C in the germ increased significantly in the early stage of germination, and it increased during the whole germination process, whereas vitamin C in the cotyledons decreased significantly in the early stage of germination, and then decreased gradually. This result shows that vitamin C in cotyledons can be transferred to germ during germination to promote the growth of germ [58].

### 4.6. Kinetic Changes of GABA in Chinese Chestnuts during Germination

GABA is a non-protein amino acid with a significant impact on human brain development. It is synthesized through the decarboxylation of glutamic acid catalyzed by glutamate decarboxylase. Subsequently, under the action of GABA transaminase, GABA is decomposed and metabolized into succinic semialdehyde, which further enters the tricarboxylic acid cycle through dehydrogenation. The higher GABA content in germs compared to cotyledons suggests that GABA is predominantly distributed in germs rather than cotyledons [25].

The GABA content in soybeans peaked at 0.89 mg/g after 12 h of germination, 4.78 mg/g in black beans at 24 h, 3.93 mg/g in green beans, and 3.09 mg/g in red beans at 36 h, respectively [59]. In contrast to annual crop seeds, Chinese chestnuts require a longer duration for the GABA content to reach its peak during germination. The GABA content in germs reached a maximum of 1.677 mg/g dry weight on the eighth day, while the GABA content in cotyledons peaked at 0.308 mg/g dry weight on the sixth day after sowing. This extended time frame may be attributed to the activation of proteolytic enzymes during germination, leading to the breakdown of proteins into free amino acids, particularly glutamic acid, which is then converted into GABA compounds [26]. The sharp decrease in GABA content in germ and cotyledon at the late stage of germination indicates the decrease in nutrient exchange efficiency between germ and cotyledon at the late stage of germination [60].

### 4.7. Kinetic Changes of Total Polyphenols and Flavonoids in Chinese Chestnuts during Germination

Polyphenols are bioactive compounds known for their diverse physiological effects, including antioxidant, anti-inflammatory, and anti-tumor properties, making them key antioxidant components in Chinese chestnuts. Flavonoids, another group of important antioxidant compounds, exhibit various physiological functions such as antibacterial, antiviral, anticancer, and anti-inflammatory activities [61]. The study results indicated that the levels of total polyphenols and flavonoids were significantly higher in germs compared to cotyledons. This disparity can be attributed to the high activity of phenylalanine aminotransferase (PLA) in the chestnut embryo, resulting in a substantial amount of enzymes that contribute to the elevated polyphenol content in germs relative to cotyledons [62].

The total phenol content in cotyledons rose during germination. Especially from day 4 to day 6, the content increased sharply. This phenomenon can be attributed to two main factors. Firstly, it is linked to the enzymatic hydrolysis of bound polyphenols, which enhances the quantity of free or extractable polyphenols. Secondly, germination induces starch breakdown and elevates glucose levels. Within the cell’s endoplasmic reticulum, glucose serves as the primary precursor for the synthesis of new polyphenol compounds through essential molecular signaling pathways, such as the pentose phosphate oxidation pathway [63]. Conversely, the gradual decrease in polyphenol content in germ may be due to the rapid increase in germ biomass.

Throughout the entire germination process, the levels of flavonoids in both germs and cotyledons exhibited a continuous increase. This phenomenon can be attributed to the enhanced accumulation of flavonoids correlating with the rise in phenylalanine ammonia-lyase (PAL) activity during germination. The conversion of trans-cinnamic acid via phenylpropane metabolism yields intermediate products such as coumaric acid, ferulic acid, and p-coumaroyl-CoA, which can further undergo transformations leading to the production of coumarin, chlorogenic acid, and coenzyme A esters, ultimately culminating in the synthesis of flavonoids and other secondary metabolite compounds [64,65].

### 4.8. Kinetic Changes of Antioxidant Activity in Chinese Chestnuts during Germination

The assessment of antioxidant activity in plant samples typically involves measuring the scavenging capabilities of DPPH and ABTS free radicals. The findings indicated that the germs exhibited significantly higher DPPH and ABTS free radical scavenging activity compared to cotyledons. This is mainly because the germ needs a higher antioxidant capacity to ensure the safety of its genetic genes.

Our research findings revealed a decrease in the anti-free radical activity of DPPH in the Chinese chestnut germ following germination. Similar results were reported by Donkor et al. in their study on sorghum germination, where they observed a decline in DPPH anti-free radical activity post-germination [66]. Conversely, Singh et al. reported an increase in DPPH inhibitory activity during sorghum germination [67]. The variance in antioxidant activity may be attributed, in part, to the chemical composition and structure of the extracted components. Indeed, the antioxidant activity of an extract is not solely dependent on its total phenol content; rather, the composition and chemical structure of its components play a significant role in determining the extract’s biological activity [68,69]. During the whole germination process, the anti-free radical activity of DPPH in cotyledons remained basically unchanged, indicating that germination had little effect on the anti-free radical activity of DPPH in Chinese chestnut cotyledons.

Furthermore, the ABTS free radical scavenging activity of germs post-germination surpassed that observed pre-germination, with peak activity noted on the 2nd and 4th days. After 4 days, the radical scavenging activity of ABTS decreased significantly, but it was still higher than that of germ per-germination. This is basically consistent with the research results of Arouna et al. [70]. On the other hand, the anti-free radical activity of DPPH in cotyledons remained basically unchanged, indicating that germination had little effect on the anti-free radical activity of ABTS in cotyledons. These findings support the notion that germination enhances antioxidant activity, highlighting sprouts of the Chinese chestnut as a valuable source of antioxidant food.

## 5. Conclusions

The data from this study clearly indicate that seed germination represents a promising approach for the production of novel functional food ingredients utilizing Chinese chestnuts. Biochemical reactions occurring during germination significantly reduced amylose, amylopectin, total sugar, and vitamin C in the cotyledons, while enhancing soluble protein, vitamin C, GABA, polyphenols, flavonoids, and ABTS antioxidant activity in Chinese chestnut germs. Furthermore, the levels of glucose, D-fructose, inositol, vitamin C, GABA, polyphenols, flavonoids, and DPPH and ABTS antioxidant activity in Chinese chestnut germs were notably higher compared to those in the cotyledons. Furthermore, the presence of D-xylulose, D-galacturonic acid, and D-ribose is characteristic of Chinese chestnut germs. Overall, Chinese chestnut germs germinated at 22 °C for 8 days represent the most suitable raw material for the development of functional food products. Our research findings highlight the Chinese chestnut and its derivatives as valuable ingredients for creating innovative foods with enhanced health benefits. Future studies will be essential to determine the optimal germination conditions for producing Chinese chestnut products with superior biological properties compared to raw kernels.

## Figures and Tables

**Table 1:** Comparison of sugar components in different parts of Chinese chestnuts during germination.

Parts	Sugar Compounds	Time (d)					
		0	2	4	6	8	10
Germ	Phenylglucoside	0.0094 ± 0.0004 ^b^	0.0085 ± 0.0003 ^c^	0.0079 ± 0.0003 ^c^	0.0065 ± 0.0003 ^e^	0.0072 ± 0.0003 ^d^	0.0126 ± 0.0005 ^a^
	Cellobiose	0.1034 ± 0.0041 ^a^	0.0948 ± 0.0038 ^b^	0.0914 ± 0.0037 ^b^	0.0656 ± 0.0026 ^d^	0.1059 ± 0.0042 ^a^	0.0749 ± 0.0030 ^c^
	Sucrose	92.5543 ± 3.7022 ^a^	89.5666 ± 3.5826 ^a^	83.3691 ± 3.3347 ^b^	68.2953 ± 2.7318 ^c^	58.1718 ± 2.3268 ^d^	52.4874 ± 2.0995 ^e^
	Maltose	0.3990 ± 0.0159 ^b^	0.3625 ± 0.0144 ^c^	0.3391 ± 0.0135 ^c^	0.3059 ± 0.0122 ^d^	0.4302 ± 0.0172 ^a^	0.1706 ± 0.0068 ^e^
	Trehalose	0.2011 ± 0.0080 ^a^	0.1916 ± 0.0076 ^ab^	0.1817 ± 0.0072 ^bc^	0.1566 ± 0.0062 ^d^	0.1740 ± 0.0069 ^c^	0.1314 ± 0.0053 ^e^
	2-Acetamido-2-deoxy-D-glucopyranose	0.0385 ± 0.0015 ^a^	0.0355 ± 0.0014 ^b^	0.0352 ± 0.0014 ^b^	0.0358 ± 0.0014 ^b^	0.0302 ± 0.0012 ^c^	0.0206 ± 0.0008 ^d^
	D-Sorbitol	0.1722 ± 0.0068 ^e^	0.2682 ± 0.0107 ^d^	0.2892 ± 0.0115 ^c^	0.4199 ± 0.0167 ^a^	0.3229 ± 0.0129 ^b^	0.1520 ± 0.0061 ^f^
	Xylitol	0.0095 ± 0.0003 ^b^	0.0087 ± 0.0003 ^c^	0.0082 ± 0.0003 ^c^	0.0073 ± 0.0002 ^d^	0.0083 ± 0.0003 ^c^	0.0102 ± 0.0004 ^a^
	D-Xylose	0.2419 ± 0.0096 ^a^	0.2172 ± 0.0086 ^b^	0.1954 ± 0.0078 ^c^	0.1653 ± 0.0066 ^d^	0.2561 ± 0.0102 ^a^	0.0997 ± 0.0039 ^e^
	D-Xylulose	0.0041 ± 0.0001 ^e^	0.0047 ± 0.0001 ^d^	0.0056 ± 0.0002 ^c^	0.0065 ± 0.0002 ^b^	0.0078 ± 0.0003 ^a^	0.0029 ± 0.0001 ^f^
	D-Galacturonic acid	0.0159 ± 0.0006 ^a^	0.0143 ± 0.0005 ^b^	0.0136 ± 0.0005 ^bc^	0.0128 ± 0.0005 ^cd^	0.0124 ± 0.0004 ^d^	0.0078 ± 0.0003 ^e^
	D-Ribose	0.0417 ± 0.0016 ^e^	0.0465 ± 0.0018 ^d^	0.0500 ± 0.0020 ^d^	0.0545 ± 0.0021 ^c^	0.0696 ± 0.0027 ^a^	0.0595 ± 0.0023 ^b^
	Barium D-ribose-5-phosphate	0.0624 ± 0.0024 ^bc^	0.0644 ± 0.0024 ^bc^	0.0648 ± 0.0025 ^ab^	0.0602 ± 0.0024 ^c^	0.0689 ± 0.0027 ^a^	0.0276 ± 0.0011 ^d^
	L-Rhamnose	0.0176 ± 0.0007 ^a^	0.0179 ± 0.0007 ^a^	0.0178 ± 0.0007 ^a^	0.0172 ± 0.0006 ^a^	0.0167 ± 0.0006 ^a^	0.0125 ± 0.0005 ^b^
	D-Mannose-6-phosphate sodium salt	0.1485 ± 0.0059 ^b^	0.1366 ± 0.0054 ^c^	0.1240 ± 0.0049 ^d^	0.1324 ± 0.0052 ^cd^	0.1577 ± 0.0063 ^a^	0.0401 ± 0.0016 ^e^
	D-Mannose	0.0644 ± 0.0025 ^e^	0.0760 ± 0.0030 ^d^	0.0851 ± 0.0034 ^c^	0.1146 ± 0.0045 ^b^	0.1259 ± 0.0050 ^a^	0.1300 ± 0.0052 ^a^
	Levoglucosan	0.5264 ± 0.0210 ^a^	0.4905 ± 0.0196 ^b^	0.4595 ± 0.0183 ^c^	0.3218 ± 0.0128 ^d^	0.3469 ± 0.0138 ^d^	0.1648 ± 0.0065 ^e^
	Inositol	9.6263 ± 0.3850 ^a^	9.1316 ± 0.3652 ^ab^	8.8565 ± 0.3542 ^b^	7.8061 ± 0.3122 ^c^	7.7569 ± 0.3102 ^c^	5.7558 ± 0.2302 ^d^
	D-Glucuronic acid	0.0618 ± 0.0024 ^e^	0.0861 ± 0.0034 ^d^	0.1142 ± 0.0045 ^c^	0.1784 ± 0.0071 ^a^	0.1704 ± 0.0068 ^a^	0.1430 ± 0.0057 ^b^
	Glucose	81.6451 ± 3.2658 ^a^	78.0234 ± 3.1209 ^ab^	75.5457 ± 3.0218 ^bc^	72.3872 ± 2.8954 ^cd^	67.5278 ± 2.7011 ^d^	41.9263 ± 1.6770 ^e^
	D-Galactose	0.2651 ± 0.0106 ^c^	0.2867 ± 0.0114 ^bc^	0.2965 ± 0.0118 ^b^	0.3052 ± 0.0122 ^b^	0.3145 ± 0.0125 ^b^	0.6600 ± 0.0264 ^a^
	L-Fucose	0.0261 ± 0.0010 ^e^	0.0289 ± 0.0011 ^d^	0.0308 ± 0.0012 ^cd^	0.0330 ± 0.0013 ^bc^	0.0352 ± 0.0014 ^b^	0.0433 ± 0.0017 ^a^
	D-Fructose	54.2171 ± 2.1686 ^a^	51.1165 ± 2.0446 ^ab^	49.4387 ± 1.9775 ^bc^	48.2138 ± 1.9285 ^bc^	46.3311 ± 1.8532 ^c^	35.1437 ± 1.4057 ^d^
	D-Arabinose	0.0840 ± 0.0033 ^a^	0.0729 ± 0.0029 ^b^	0.0605 ± 0.0024 ^c^	0.0567 ± 0.0022 ^cd^	0.0550 ± 0.0022 ^d^	0.0270 ± 0.0010 ^e^
	D-Arabinitol	0.0158 ± 0.0006 ^b^	0.0163 ± 0.0006 ^ab^	0.0167 ± 0.0006 ^ab^	0.0175 ± 0.0007 ^a^	0.0170 ± 0.0006 ^ab^	0.0123 ± 0.0004 ^c^
	D-Ribono-1,4-lactone	0.0073 ± 0.0002 ^d^	0.0086 ± 0.0003 ^c^	0.0097 ± 0.0003 ^b^	0.0108 ± 0.0004 ^a^	0.0108 ± 0.0004 ^a^	0.0083 ± 0.0003 ^c^
	Raffinose	0.4615 ± 0.0184 ^a^	0.3886 ± 0.0155 ^b^	0.3639 ± 0.0145 ^c^	0.3056 ± 0.0122 ^d^	0.2424 ± 0.0096 ^e^	0.1741 ± 0.0069 ^f^
Cotyledon	Phenylglucoside	0.0064 ± 0.0002 ^a^	0.0056 ± 0.0002 ^b^	0.0057 ± 0.0002 ^b^	0.0046 ± 0.0001 ^d^	0.0050 ± 0.0002 ^c^	0.0041 ± 0.0002 ^e^
	Cellobiose	0.1134 ± 0.0045 ^b^	0.1345 ± 0.0053 ^a^	0.1159 ± 0.0046 ^b^	0.0723 ± 0.0028 ^d^	0.0623 ± 0.0024 ^e^	0.0796 ± 0.0031 ^c^
	Sucrose	119.2855 ± 4.7714 ^a^	118.9857 ± 4.7594 ^a^	118.9038 ± 4.7561 ^a^	117.6958 ± 4.7078 ^a^	120.1981 ± 4.8079 ^a^	107.3171 ± 4.2926 ^b^
	Maltose	1.4680 ± 0.0587 ^b^	1.6245 ± 0.0649 ^a^	1.2986 ± 0.0519 ^c^	0.8965 ± 0.0358 ^d^	0.8491 ± 0.0339 ^d^	0.8999 ± 0.0359 ^d^
	Trehalose	0.0114 ± 0.0004 ^d^	0.0202 ± 0.0008 ^a^	0.0124 ± 0.0004 ^d^	0.0144 ± 0.0005 ^c^	0.0158 ± 0.0006 ^b^	0.0138 ± 0.0005 ^c^
	2-Acetamido-2-deoxy-D-glucopyranose	0.0401 ± 0.0016 ^a^	0.0355 ± 0.0014 ^b^	0.0331 ± 0.0013 ^c^	0.0245 ± 0.0009 ^d^	0.0336 ± 0.0013 ^bc^	0.0210 ± 0.0008 ^e^
	D-Sorbitol	0.4769 ± 0.0190 ^c^	0.6675 ± 0.0267 ^b^	0.4251 ± 0.0170 ^d^	0.3189 ± 0.0127 ^e^	0.8170 ± 0.0326 ^a^	0.4191 ± 0.0168 ^d^
	Xylitol	0.0069 ± 0.0002 ^b^	0.0091 ± 0.0003 ^a^	0.0066 ± 0.0002 ^bc^	0.0063 ± 0.0002 ^cd^	0.0064 ± 0.0002 ^bc^	0.0058 ± 0.0002 ^d^
	D-Xylose	0.0060 ± 0.0002 ^c^	0.0072 ± 0.0002 ^ab^	0.0070 ± 0.0002 ^b^	0.0076 ± 0.0003 ^a^	0.0068 ± 0.0002 ^b^	0.0073 ± 0.0003 ^ab^
	D-Xylulose	-	-	-	-	-	-
	D-Galacturonic acid	-	-	-	-	-	-
	D-Ribose	-	-	-	-	-	-
	Barium D-ribose-5-phosphate	0.0482 ± 0.0019 ^a^	0.0479 ± 0.0019 ^a^	0.0438 ± 0.0017 ^b^	0.0416 ± 0.0016 ^b^	0.0420 ± 0.0016 ^b^	0.0356 ± 0.0014 ^c^
	L-Rhamnose	0.0099 ± 0.0003 ^a^	0.0089 ± 0.0003 ^bc^	0.0089 ± 0.0003 ^bc^	0.0093 ± 0.0003 ^ab^	0.0096 ± 0.0003 ^ab^	0.0084 ± 0.0003 ^c^
	D-Mannose-6-phosphate sodium salt	0.0215 ± 0.0008 ^a^	0.0196 ± 0.0007 ^b^	0.0164 ± 0.0006 ^c^	0.0159 ± 0.0006 ^cd^	0.0165 ± 0.0006 ^c^	0.0148 ± 0.0005 ^d^
	D-Mannose	0.0275 ± 0.0011 ^d^	0.0370 ± 0.0014 ^c^	0.0287 ± 0.0011 ^d^	0.0540 ± 0.0021 ^a^	0.0347 ± 0.0013 ^c^	0.0435 ± 0.0017 ^b^
	Levoglucosan	0.0824 ± 0.0033 ^a^	0.0842 ± 0.0033 ^a^	0.0706 ± 0.0028 ^b^	0.0843 ± 0.0033 ^a^	0.0804 ± 0.0032 ^a^	0.0691 ± 0.0027 ^b^
	Inositol	1.4963 ± 0.0598 ^b^	1.7381 ± 0.0695 ^a^	0.9445 ± 0.0377 ^c^	1.4921 ± 0.0596 ^b^	1.7602 ± 0.0704 ^a^	0.9322 ± 0.0372 ^c^
	D-Glucuronic acid	0.1003 ± 0.0040 ^c^	0.1479 ± 0.0059 ^b^	0.1542 ± 0.0061 ^b^	0.1710 ± 0.0068 ^a^	0.1649 ± 0.0065 ^a^	0.0885 ± 0.0035 ^d^
	Glucose	0.7112 ± 0.0284 ^e^	1.3680 ± 0.0547 ^d^	1.7088 ± 0.0683 ^c^	2.7176 ± 0.1087 ^a^	2.1952 ± 0.0878 ^b^	1.7671 ± 0.0706 ^c^
	D-Galactose	0.0544 ± 0.0021 ^d^	0.0416 ± 0.0016 ^e^	0.0665 ± 0.0026 ^c^	0.1137 ± 0.0045 ^a^	0.0942 ± 0.0033 ^b^	0.0893 ± 0.0035 ^b^
	L-Fucose	0.0139 ± 0.0005 ^bc^	0.0149 ± 0.0005 ^ab^	0.0131 ± 0.0005 ^c^	0.0157 ± 0.0006 ^a^	0.0144 ± 0.0005 ^b^	0.0145 ± 0.0005 ^b^
	D-Fructose	1.5422 ± 0.0616 ^c^	1.6012 ± 0.0556 ^bc^	1.6178 ± 0.0647 ^bc^	3.2509 ± 0.1300 ^a^	3.2262 ± 0.1290 ^a^	1.7295 ± 0.0692 ^b^
	D-Arabinose	0.0084 ± 0.0003 ^bc^	0.0073 ± 0.0003 ^d^	0.0085 ± 0.0003 ^bc^	0.0097 ± 0.0004 ^a^	0.0088 ± 0.0004 ^b^	0.0080 ± 0.0003 ^c^
	D-Arabinitol	0.0069 ± 0.0002 ^a^	0.0069 ± 0.0003 ^a^	0.0068 ± 0.0003 ^a^	0.0069 ± 0.0003 ^a^	0.0068 ± 0.0003 ^a^	0.0067 ± 0.0003 ^a^
	D-Ribono-1,4-lactone	0.0071 ± 0.0003 ^bc^	0.0066 ± 0.0003 ^c^	0.0076 ± 0.0003 ^ab^	0.0073 ± 0.0003 ^ab^	0.0078 ± 0.0003 ^a^	0.0078 ± 0.0003 ^a^
	Raffinose	0.8876 ± 0.0355 ^b^	0.7389 ± 0.0295 ^c^	0.9823 ± 0.0392 ^a^	0.7802 ± 0.0312 ^c^	0.9798 ± 0.0392 ^a^	0.2049 ± 0.0082 ^d^

The presented results represent mean ± standard deviations, n = 3. Different letters represent significant differences among treatments for each sampling time at *p* ≤ 0.05.

## Data Availability

The original contributions presented in the study are included in the article, further inquiries can be directed to the corresponding author.
